# Evaluation of the clinical efficacy of a water soluble formulation of tylvalosin in the control of enzootic pneumonia associated with *Mycoplasma hyopneumoniae* and *Pasteurella multocida* in pigs

**DOI:** 10.1186/s40813-020-00177-9

**Published:** 2020-12-04

**Authors:** Alfonso Lopez Rodriguez, Anna Catharina Berge, Cliff Ramage, Ryan Saltzman, Rickie J. Domangue, Mark J. Gnozzio, Annika Muller, Pascale Sierra, Hafid A. Benchaoui

**Affiliations:** 1 ECO Animal Health Ltd., London, UK; 2 Berge Veterinary Consulting BV, Vollezele, Belgium; 3 Moredun Scientific, Bush Loan, Penicuik, UK; 4 Veterinary Resources Inc., Ames, IA USA; 5 Rickie J. Domangue Statistical Consulting Services, Broadway, VA USA; 6 ECO Animal Health Inc., Princeton, NJ USA; 7 Charles River Laboratories Edinburgh Ltd, Edinburgh, UK; 8 Parscivet, Gerstheim, France

**Keywords:** Tylvalosin, Macrolide, Aivlosin, *Mycoplasma hyopneumoniae*, Enzootic pneumonia, Porcine respiratory disease

## Abstract

**Background:**

The efficacy of a water soluble formulation of tylvalosin (Aivlosin® 625 mg/g granules) was evaluated in the treatment and metaphylaxis of Enzootic Pneumonia (EP) in pigs. In all four trials, pigs in the tylvalosin group were administered 10 mg tylvalosin/kg bodyweight in drinking water daily for 5 consecutive days (TVN). In a single-challenge study, pigs were inoculated with lung homogenate containing *Mycoplasma hyopneumoniae.* In a dual challenge study, pigs were sequentially inoculated with pure culture of *M. hyopneumoniae* and *Pasteurella multocida.* Efficacy was evaluated based on reduction of lung lesions compared to unmedicated control pigs (CTL)*.* In two field studies at European commercial farms with confirmed outbreaks of EP, treatment efficacy in clinically affected fatteners was evaluated based on improved clinical conditions compared to pigs treated with tylosin at 10 mg/kg by injection for 3 consecutive days (TYL). In these field trials, healthy in contact pigs were enrolled for metaphylaxis efficacy evaluation based on reduction in incidence of new clinical cases of respiratory disease compared to unmedicated pigs (CTL).

**Results:**

In the *M. hyopneumoniae*-only challenge study, pigs in TVN group had lower lung lesion scores than CTL (6.52 vs. 14.97; *p* <  0.001). In the dual challenge study with *M. hyopneumoniae* and *P. multocida*, pigs in TVN group had lower lung lesion scores than CTL (3.32 vs. 8.37; *p* <  0.01) and the recovery of both challenge bacteria from the lungs was lower in TVN compared with CTL group (*p* <  0.01). In field outbreaks of EP, multicentre analysis showed that 13 days after the start of medication, treatment success for TVN pigs was significantly better than for TYL pigs (80.0% vs 48.7% *p* = 0.03) and metaphylactic administration of TVN significantly reduced the incidence of new clinical cases (2.1% vs. 7.8%; *p* <  0.01) compared with unmedicated controls.

**Conclusions:**

Tylvalosin at 10 mg/kg daily for 5 days in drinking water was safe and effective in the treatment and metaphylaxis of EP in pigs associated with infections of *M. hyopneumoniae* either alone or in combination with *P. multocida* under both experimental challenge and field natural infection conditions.

## Background

*Mycoplasma hyopneumoniae* is the primary causative agent implicated in Enzootic Pneumonia (EP) in pigs [1, 2]. However, in field outbreaks the condition is frequently complicated by secondary bacterial infections with a range of bacterial pathogens, commonly including *Pasteurella multocida, Bordetella bronchiseptica* and *Glässerella* (formerly *Haemophilus parasuis*) [1, 2]. Enzootic Pneumonia is a major problem affecting growing pigs worldwide with a range of symptoms, including pyrexia and coughing, and the development of pneumonic lesions in the lung, leading to reductions in feed conversion efficiency and growth rates [1, 2]. The condition is multifactorial whereby factors such as stocking density and housing design, as well as environmental and climatic variables can all affect the severity of an outbreak, making effective control an ongoing challenge for clinicians and farmers. In order to control an outbreak within a unit and to maintain the welfare of affected animals, the judicious use of antimicrobials is often necessary, both to treat pigs showing clinical disease and to minimise the spread of infection to in-contact animals [1, 2]. To do this, an effective antimicrobial needs to be administered and in many cases macrolide antibiotics have been shown to be efficacious for this purpose [3–5]. However, it can be challenging to administer injectable medication to all animals in a group without inducing additional stress and worsening the clinical presentation. In this context, the use of a formulation that can be administered via the drinking water may be a preferable approach. The objective of these studies was to determine the clinical efficacy of a water-soluble formulation containing tylvalosin (Aivlosin® 625 mg/g granules for use in drinking water, ECO Animal Health) administered via drinking water at 10 mg/kg bodyweight against EP associated with *M. hyopneumoniae,* either alone or combined with *P. multocida*. Efficacy was evaluated both in experimental challenge models and also in natural infections under representative field conditions in the EU.

## Results

### Challenge studies

#### Lung lesions

**Fig. 1:**
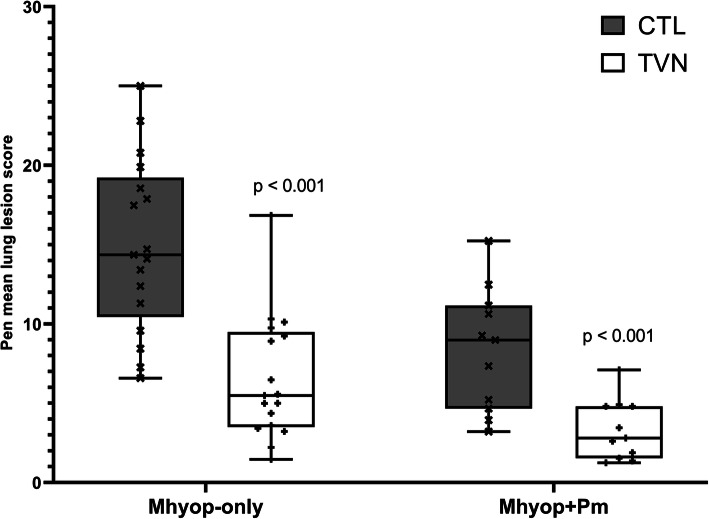
Pen mean lung lesion scores for tylvalosin pigs (TVN group) and untreated pigs (CTL group) following experimental inoculation with *M. hyopneumoniae* alone (Mhyop-only) or in a sequential combination with *P. multocida* (Mhyop + Pm). Box plots represents the arithmetic mean of each pen. In Mhyop-only, there were 6 pigs in each pen, 17 pens for each treatment. Pigs were challenged endotracheally with lung homogenate containing 1 × 10^5^ ccu of *M. hyopneumoniae* in 10 ml on Day 0 and Day 1 and scored on Day 28. In Mhyop+Pm, there were 4 pigs in each pen, 11 pens for each treatment. Pigs were challenged on Day 0, Day 1 and Day 3 with broth media containing 1 × 10^9^ cfu of *M. hyopneumoniae* in 5 ml of Mhyop and on Day 14 with broth media containing 1 × 10^10^ cfu in 10 ml of *P. multocida*. Both challenges through intranasal route and scored on Day 28. *P* value from least square means derived from statistical models

Following challenge with *M. hyopneumoniae* only (Mhyop-only study), mean lung lesion scores at necropsy on Day 28 post challenge were 56% lower for pigs treated with tylvalosin (TVN) compared with unmedicated control pigs (CTL) (6.52 vs. 14.97; *p* <  0.01) (Fig. 1).

In the dual-challenge study (Mhyop+Pm study), at necropsy on Day 28 post *M. hyopneumoniae* challenge (14 days post *P. multocida* challenge), mean lung lesion scores in TVN were 60% lower than for CTL (3.32 vs. 8.37; *p* <  0.01).

#### Microbiological recovery

**Fig. 2:**
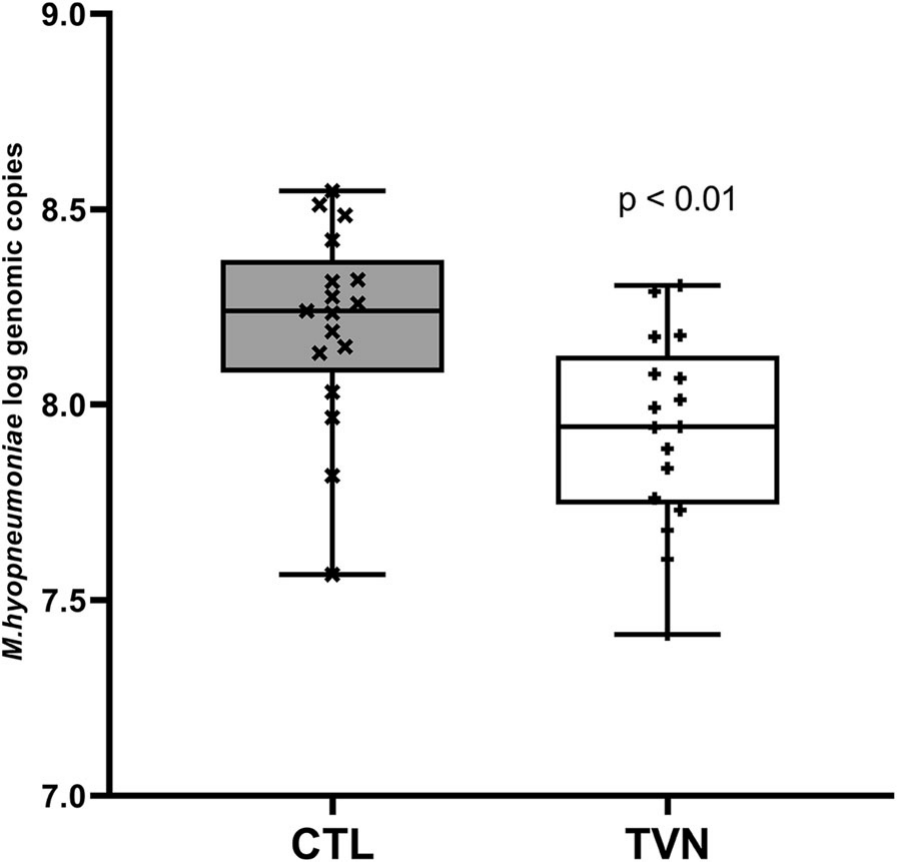
Pen mean *M. hyopneumoniae* genomic copies (log transformed) in bronchoalveolar lavage recovered at necropsy in *M. hyopneumoniae* only challenge study (Mhyop-only) for tylvalosin pigs (TVN group) and untreated pigs (CTL group). Box plots represents the arithmetic mean of each pen. There were 6 pigs in each pen, 17 pens for each treatment. Pigs were challenged endotracheally with lung homogenate containing 1 × 10^5^ ccu of *M. hyopneumoniae* in 10 ml on Day 0 and Day 1 and samples collected on Day 28. *P* values from least square means derived from statistical models

In the Mhyop-only challenge study, the *M. hyopneumoniae* DNA copies by polymerase chain reaction (PCR) in bronchoalveolar lavage (BAL) collected at necropsy were significantly lower for TVN than for CTL (*p* < 0.01; Fig. 2).

**Fig. 3:**
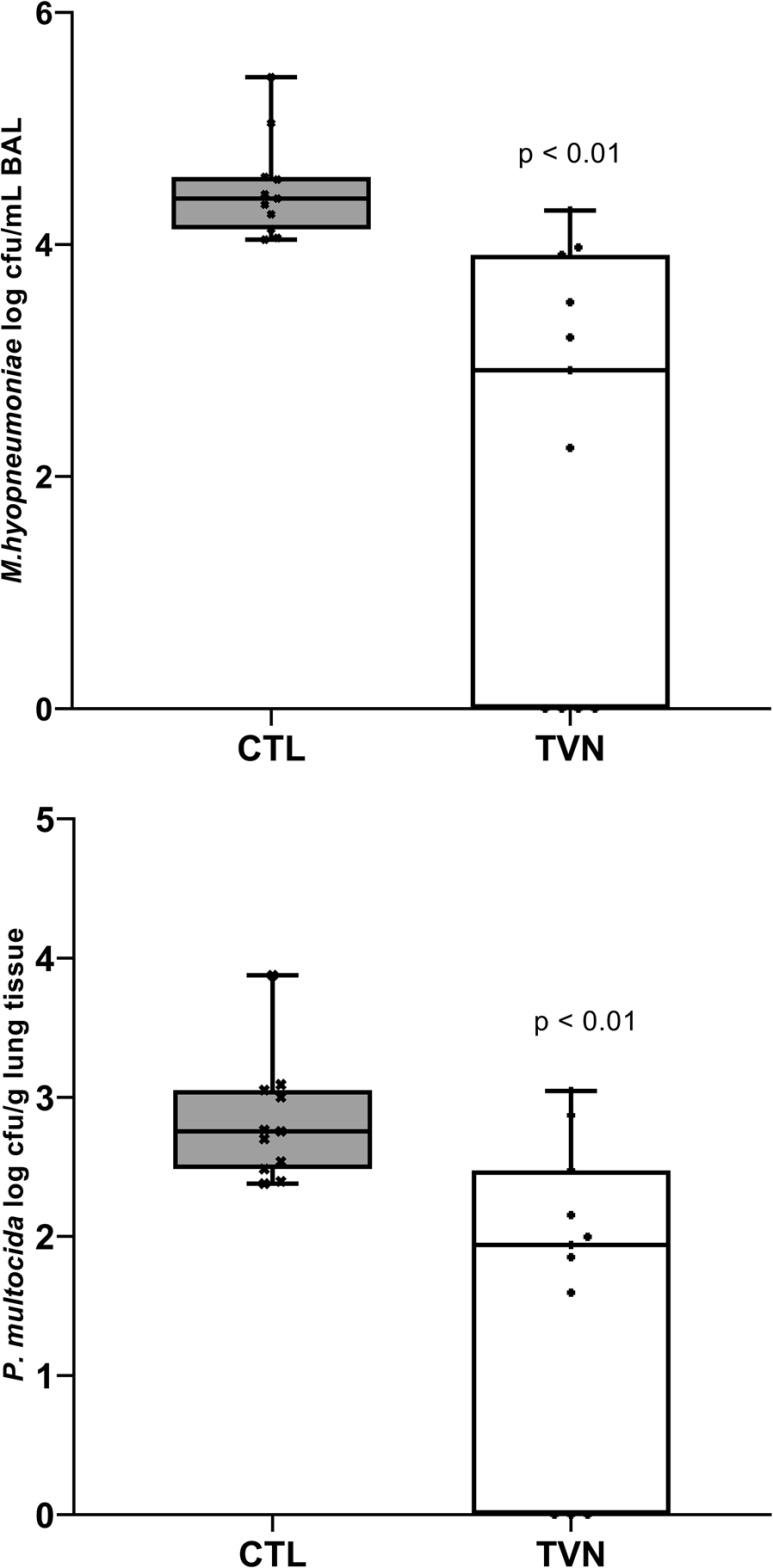
Log transformed pen mean *M. hyopneumoniae* colony forming units (cfu) in bronchoalveolar lavage and log transformed pen mean *P. multocida* cfu in lung tissue recovered at necropsy in Mhyop + Pm dual-challenge study for tylvalosin pigs (TVN group) and untreated pigs (CTL group). Box plots represents the arithmetic mean of each pen. There were 4 pigs in each pen, 11 pens for each treatment. Pigs were challenged on Day 0, Day 1 and Day 3 with broth media containing 1 × 10^9^ cfu of *M. hyopneumoniae* in 5 ml of Mhyop and on Day 14 with broth media containing 1 × 10^10^ cfu in 10 ml of *P. multocida*. Both challenges through intranasal route and scored on Day 28. P values from least square means derived from statistical models

In the Mhyop+Pm challenge study, the mean recovery of *M. hyopneumoniae* by culture from BAL collected at necropsy was lower for TVN than CTL (*p* < 0.01; Fig. 3) and all pigs were negative in 4 out of 11 pens in TVN whereas all pens in CTL had at least one pig positive (Fig. 3). Furthermore, significantly more pigs (70.5%) in TVN were negative on BAL culture for *M. hyopneumoniae* compared to only 2.3% of pigs in CTL (*p* < 0.01). In this Mhyop+Pm dual challenge study, the mean *P. multocida* count was lower in TVN compared with CTL (*p* < 0.01; Fig. 3) and there was no isolation of *P. multocida* in 3 of 11 pens in TVN, whereas all pens in CTL had at least one pig positive (Fig. 3). Furthermore, significantly more pigs (70.5%) in TVN were negative on culture for *P. multocida* compared to only 29.5% of pigs in CTL (*p* < 0.01).

#### Clinical observations

In the Mhyop-only challenge study, pigs in TVN coughed significantly less than those in CTL with least squares mean scores of 11.1 vs. 23.5, respectively (*p* < 0.01).

In the Mhyop+Pm challenge study, demeanour, nasal discharge or coughing were virtually absent in all animals and no significant differences were found between the two groups. Mean respiration scores were numerically lower in TVN than CTL (0.15 vs. 0.21; *p* = 0.07).

#### Weight gain and feed intake parameters

Daily weight gain in the Mhyop-only challenge study was improved in TVN compared to CTL (0.72 vs. 0.64 kg/pig/day; *p* < 0.01). Mean feed consumption was also improved in TVN compared to CTL (1.19 vs. 1.12 kg/day; *p* = 0.02) and feed conversion ratio was significantly better in TVN pigs compared to CTL pigs (1.66 vs. 1.73; *p* = 0.02).

In the Mhyop+Pm challenge study, average daily weight gain was numerically but not significantly higher for TVN than CTL pigs (0.62 vs. 0.55 kg/pig/day; *p* = 0.11). There were no differences in daily feed intake but the feed conversion ratio was significantly better in TVN compared to CTL pigs (1.57 vs. 1.83; *p* < 0.01).

### Field studies

#### Treatment efficacy

Treatment success was significantly higher for TVN compared with TYL (*p* = 0.03) when data were combined across the study sites (Table 1). In Hungary, TVN treatment success was not significantly higher than TYL (*p* = 1.00), whereas in France treatment success rate was significantly higher for TVN than TYL (Table 1; *p* = 0.01). The non-inferiority of TVN to TYL was confirmed in both sites as well as in combined data analysis (Table 1).

**Table 1:** Treatment efficacy of tylvalosin (TVN) and tylosin (TYL) against natural outbreaks of enzootic pneumonia associated with *M. hyopneumoniae* in Hungary and France

Study	***n***	Day 0 clinical cases ***n*** (% of total)	Treatment success on study completion^**a**^		
			***n*** (%)	Group comparison ***p-***values	Non inferiority p-values^**b**^
**Hungary**					
TVN	255	12 (4.7%)	11 (91.7%)	1.00	0.04
TYL	241	15 (6.2%)	13 (86.7%)		
**France**					
TVN	183	18 (9.8%)	13 (72.2%)	0.01	< 0.01
TYL	182	24 (13.2%)	6 (25.0%)		
**Both sites combined**					
TVN	438	30 (6.8%)	24 (80.0%)	0.03	< 0.01
TYL	423	39 (9.2%)	19 (48.7%)		

*M. hyopneumoniae* and other bacteria including *P. multocida* were isolated at both sites prior to Day 0

*n* Number of pigs

^a^Absence of treatment failures and relapses through to Day 13

^b^Non-inferior to control product (Farrington Manning *p* ≤ 0.05 indicates non inferiority to control product)

#### Metaphylactic efficacy

From treatment completion to study end, the risk of becoming clinically ill was significantly lower in TVN pigs compared to CTL pigs in Hungary (*p* = 0.03) and in France (*p* = 0.02) (Table 2). The combined data from the two sites also showed that the risk of becoming clinically ill was lower in TVN pigs compared to untreated animals (*p* < 0.01; Table 2).

**Table 2:** Metaphylactic efficacy of tylvalosin (TVN) compared with unmedicated controls (CTL) during natural outbreaks of enzootic pneumonia associated with *M. hyopneumoniae* in Hungary and France

	Healthy on			Frequency of new cases	
	Day 0	Day 5	Day 13	Day 5–13	
	***n***	***n***	***n***	***n*** (%)	***p***-value*
**Hungary**					
TVN	239	226	224	2 (0.9%)	0.03
CTL	219	201	192	9 (4.5%)	
**France**					
TVN	165	111	106	5 (4.5%)	0.02
CTL	157	106	91	15 (14.2%)	
**Both sites combined**					
TVN	404	340	330	7 (2.1%)	< 0.01
CTL	376	307	283	24 (7.8%)	

*M. hyopneumoniae* and other bacteria including *P. multocida* were isolated at both sites prior to Day 0

* *p*-value from logistic model with random effect of pen in each site and random effect of site in combined analysis

**Fig. 4:**
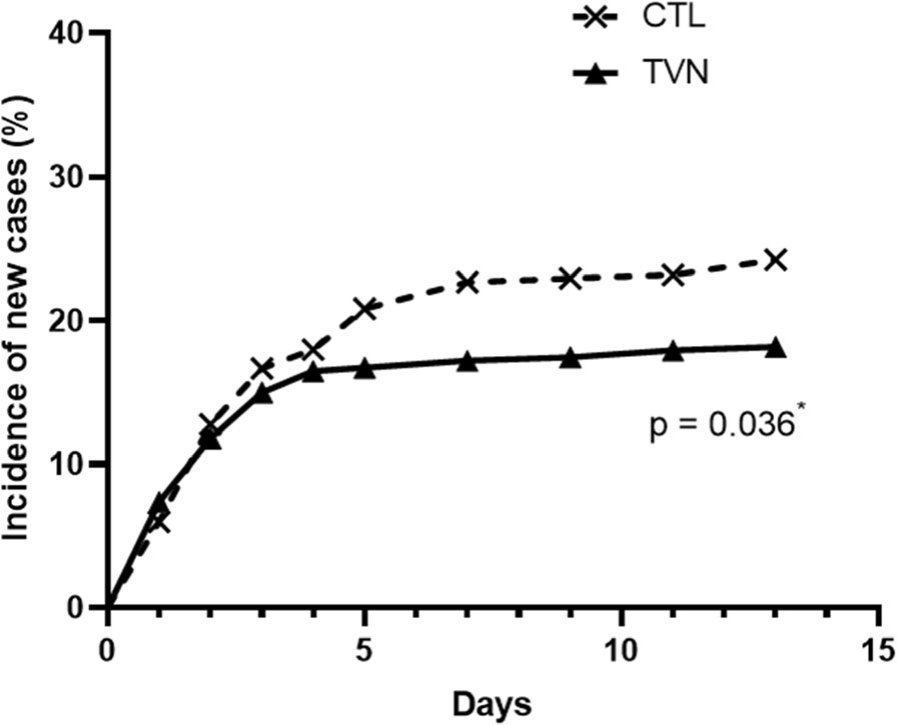
Incidence of new clinical cases for tylvalosin pigs (TVN group) and untreated pigs (CTL group) in the healthy population (data of 2 sites combined). Pigs in the TVN pens received Aivlosin in drinking water at 10 mg tylavlosin/kg body weight daily for 5 consecutive days. Pigs in the CTL pens were left untreated. A pig was clased as new case if it was deemed healthy at the start of the evaluation period but met the clinically affected criteria (see Table 4) up to Day 13. ^*^Log-rank *P*-value from testing homogeneity of survival curves for event day over strata indicates more new cases in untreated pigs

From Day 1 to Day 13, the survival analysis log-rank statistics on combined data of both sites showed that the percentage of pigs becoming ill was significantly higher for CTL pigs than for TVN pigs (Fig. 4; *p* = 0.04).

#### Safety

No drug related adverse events occurred in any pigs during these 4 studies and no injection site reactions were observed in any animal following injection with TYL or any rescue medication in the field studies.

## Discussion

Tylvalosin is known to have efficacy against *M. hyopneumoniae* and the premix formulation (Aivlosin® 42.5 mg/g premix for medicated feeding stuff) and oral powder formulation (Aivlosin® 42.5 mg/g oral powder for pigs) of tylvalosin are licensed in the EU for the treatment and metaphylaxis of EP [3]. Recently, the Committee for Veterinary Medicinal Products (CVMP) granted approval for the water-soluble formulation (Aivlosin® 625 mg/g granules for use in drinking water) for the same indication against EP. The studies summarised in this article were undertaken to determine the efficacy against EP of the water-soluble formulation at the dose rate of 10 mg tylvalosin/kg bodyweight.

Infection with *M. hyopneumoniae* results in lung lesions and mild clinical disease that may impact the growth and feed conversion efficiency of affected pigs, as was observed in the challenge studies even in relatively short time periods. In cases of EP, combined infections with secondary bacteria are common and tend to result in a more severe pneumonia and clinical disease [1, 2]. *P. multocida* is one of the most common secondary pathogens isolated from cases of EP [6]. In order to mimic EP, pigs were inoculated concurrently with *P. multocida* in one of the two experimental models reported here.

Challenge studies were successful as they induced lung lesions and clinical disease comparable to published literature [7–11]. It is known that clinical disease is generally mild in both single and dual infection challenge models and therefore the key parameters for measurement of efficacy against EP in challenge studies were lung lesions and recovery of infectious agents (*M. hyopneumoniae* and *P. multocida*) [7–11]. Both variables were significantly improved by tylvalosin, indicating good efficacy. Lower lung lesion scores were observed in pigs in the combined challenge study compared to the pigs in the Mhyop-only study. It has been previously shown that the intranasal route used in the Mhyop+Pm study results in lower lesions than the endotracheal route used in the Mhyop-only study [12]. Other factors that could explain this difference are the challenge strain and possibly the use of lung homogenate rather than pure culture as the challenge material [13]. Regardless of the challenge model, the relative improvement compared to the control group (~ 60%) was similar in both studies, indicating consistent efficacy.

Recent data indicates that susceptibility of *M. hyopneumoniae* to tylvalosin is high and that tylvalosin can be bactericidal at concentrations above MIC [14]. The expected efficacy based on previously obtained MIC data was confirmed in the studies summarised in this article. A potential bactericidal effect in vivo is supported by the lower recovery of *M. hyopneumoniae* from the lungs compared to untreated pigs in both challenge studies, four weeks after infection and 8–9 days after treatment.

There are no published data on susceptibility of *P. multocida* to tylvalosin but the MIC value for tylvalosin against the strain used in the Mhyop+Pm dual challenge study was very high (≥ 128 μg/ml). Despite this, clinical efficacy was demonstrated by reduction in lung lesions and bacterial recovery in the Mhyop+Pm dual challenge study. Previous reports have suggested that in vitro susceptibility is not always predictive of in vivo efficacy because the methodology used in the sensitivity testing may affect the results [15]. A disconnect between MIC and clinical efficacy has been previously reported for macrolides against respiratory pathogens in calves and swine [16, 17] and this disconnect is reinforced by data presented here.

Furthermore, it is well known that macrolides, including tylvalosin, have immunomodulatory effects which may impact in vivo efficacy [18, 19]. Increasing evidence demonstrates that tylvalosin exerts such effects by increasing the apoptosis of porcine neutrophils and macrophages, increasing the release of Lipoxin A4 and Resolvin D1, while inhibiting the production and release of pro-inflammatory Leukotriene B4, Interleukin-8 and Interleukin-1α [18]. These highly complex interactions may have aided the fight against disease.

Typically, infection with *M. hyopneumoniae* is a chronic problem within a commercial unit, often spreading slowly but causing associated clinical signs and marked production losses [20, 21]. However, studies with seeder pigs have shown that infection can spread more rapidly, within just 14 days of initial exposure, although this may not result in overt clinical signs in all affected pigs [9]. The use of vaccination or high health status disease free pigs may allow for a degree of control of respiratory disease associated with *M. hyopneumoniae* [1, 22]. However, these approaches are not always successful in preventing disease as observed in these field studies where EP outbreaks were declared despite vaccination. To maintain welfare, affected pigs need to be treated with an effective antimicrobial [1]. In the challenge studies, the microbiological results showed a clear benefit to general health of tylvalosin compared with placebo. In the context of naturally occurring infections, reducing the prevalence of *M. hyopneumoniae* infection could be expected to markedly decrease subsequent episodes of respiratory disease amongst the pigs sharing the same air space and physical contact. The field studies were undertaken in order to test this hypothesis.

From a judicious use perspective, initiation of treatment in the field studies was only undertaken once EP had been diagnosed and after at least 30% of the pens had at least 10% animals that were clinically affected and intervention was justified. Diagnosis of EP in the field was based on clinical signs, epidemiology and typical lesions in previous batches of animals, and confirmation of *M. hyopneumoniae* infection by PCR [2]. The value of PCR testing in determining the presence of specific infective agents was confirmed in these field studies. Its principal advantage is to avoid the difficult and time consuming exercise of culturing fastidious organisms from imperfect sampling. Unfortunately, the results of oral fluid samples collected from ropes hung in the pens proved to be extremely erratic and provided no usable data for efficacy evaluation. This highlighted the limited value of this technique in these studies, probably due to the inconsistency with which animals chewed on the ropes and potentially complicated by inconsistent excretion of pathogens in saliva by infected pigs [23]. However, they were useful to demonstrate the presence of infection. Considering the low sensitivity of the test, the fact that oral fluids of several pens tested positive is indicative of high circulation of *M. hyopneumoniae* in the unit [23].

Commercially available formulations of tylvalosin and injectable tylosin were used, and the control product was used in accordance with the authorised directions for use at the highest approved dose rate for the condition. There is no clear explanation for the poor treatment success rate for pigs treated with tylosin in France. The isolates of *M. hyopneumoniae* tested were susceptible to tylosin at both sites and no protocol deviations were reported for administration of tylosin. The level of viral infection circulating within the barn may partially explain lack of efficacy, but it would be expected to have affected both groups equally. Altogether, field data supported a better clinical efficacy of tylvalosin administered in drinking water for 5 day than an injectable formulation of tylosin for 3 days for the treatment and metaphylaxis of EP.

The field data shows that disease progressed similarly in the first 2–3 days of the outbreak in untreated pigs and TVN. Animals developing clinical signs during the first days of the outbreak were almost certainly incubating the disease prior to or at start of medication. Therefore, the outcome of the intervention measured on completion of the full course of treatment is a useful parameter to evaluate metaphylaxis efficacy. The field studies show that once the drug is given sufficient time to exert its full effect, the disease continued to progress at a higher rate in the unmedicated pigs than those in TVN.

## Conclusions

The studies presented have demonstrated that tylvalosin administered via drinking water at 10 mg/kg was safe and effective in the treatment and metaphylaxis of EP associated with *M. hyopneumoniae* with or without concurrent bacterial infections (notably *P. multocida*) both under controlled experimental conditions and those commonly encountered in the field.

## Materials and methods

All studies were conducted in accordance with the principles of Good Clinical Practice (GCP) and followed a randomised, controlled, blinded, parallel study design [24].

All studies were masked by person. Personnel assessing efficacy variables were masked as to treatment allocation, and this masking was maintained throughout; personnel involved in administering the medications were unmasked and did not participate in any efficacy observations.

For all studies, pigs in the TVN group received tylvalosin administered via the drinking water at the nominal daily dose rate of 10 mg/kg bodyweight for five consecutive days. In the field studies, clinically affected pigs in the TYL group received tylosin (Pharmasin®, Huvepharma NV) administered intramuscularly (IM) at 10 mg/kg for three consecutive days. Animals that were treatment failures in the field or that relapsed after completion of the course of treatment received a single IM injection of tulathromycin (Draxxin® 100, Zoetis) at 2.5 mg/kg as a rescue therapy.

### Animal husbandry & housing

In the series of two challenge studies, commercial Landrace/Large White/Duroc pigs (aged 3–5 weeks old) were confirmed negative for *M. hyopneumoniae* antibodies by enzyme linked immuno-sorbent assay (ELISA) testing prior to challenge. In the Mhyop-only challenge study, a subsample of 36 randomly selected pigs (~ 17%) were also tested for *M. hyopneumoniae* by PCR on nasal swabs and all samples were negative. Enrolled animals had no clinical signs of other respiratory, enteric or systemic disease. Pigs were housed in multiple separate pens in semi-containment accommodation sharing the same airspace. In the Mhyop-only challenge study, each treatment group consisted of 102 pigs housed in 17 pens with 6 pigs each. In the Mhyop+Pm study, there were 11 pens with four pigs each in each treatment group (*n* = 44 pigs per group). The accommodations were cleaned and thoroughly disinfected prior to introduction of the piglets. The accommodations had artificial lighting and ventilation and were maintained at an appropriate temperature using wall fan heaters and additional heat lamps if required. Animals were fed a suitable commercially available antibiotic-free pig feed twice daily at an appropriate rate for animals of this age. Water was provided ad libitum.

In the field studies, typical commercial premises were utilised, with the pre-existing accommodation with slatted floors and feeding regimes. A total of 496 pigs were enrolled in a site in Hungary and 365 pigs in a site in France. Within each house, pens (22 pens each containing 22–24 pigs in Hungary or 36 pens each containing 9–11 pigs in France) were supplied with water from two different water lines. Each water line was connected to a proportioner system to allow administration of either medicated or non-medicated water in accordance with the pre-determined allocation plan. Water lines were mounted in chess-board pattern to ensure that pens of both groups were equally distributed across the pig unit.

### Laboratory efficacy challenge studies design

In the Mhyop-only challenge study, pigs were inoculated with lung homogenate containing approximately 1 × 10^5^ ccu of a well characterised *M. hyopneumoniae* strain in 10 ml [25]. Each pig was given 10 ml of challenge endotracheally on Days 0 and 1. Animals in the TVN group were given medicated water daily for 5 days at the onset of coughing (from Day 14 to 19). All animals were euthanized on Day 28.

In the Mhyop+Pm dual challenge study, each pig was inoculated with broth media containing approximately 1 × 10^9^ cfu of *M. hyopneumoniae* in 5 ml on Days 0, 1 and 2 and approximately 1 × 10^10^ cfu of *P. multocida* in 10 ml on Day 14, both recently isolated in the UK. Inoculations were given intranasally using a syringe and an aerosol adapter, with approximately half of the challenge material administered to each nostril. Animals in the TVN group were given medicated water daily for 5 days at the onset of clinical signs (from Day 15 to 20). All animals were euthanised on Day 28.

In vitro minimum inhibitory concentrations (MICs) for *M. hyopneumoniae* were determined by standard broth microdilution MIC methodology specifically modified using Friis medium for growing Mycoplasma species and Mueller Hinton broth for bacteria [26, 27]. MICs for tylvalosin against these challenge strains were 0.008 μg/ml for 232MP and 0.015 μg/ml for 42P11. The MIC for tylvalosin against *P. multocida* Y05576 was > 128 μg/ml.

In both studies, animals were observed daily for general health and clinical signs of respiratory disease. Rectal temperatures were recorded and animals were weighed at the start of medication and at study end. Observations were used to ascribe clinical scores in accordance with the categorisation shown in Tables 3 and 4. Body weights were used to calculate daily weight gain as the body weight at the study end minus the body weight at the start of medication divided by the number of days in that period. At examination post mortem, samples of lung lavage and lung tissue were collected for microbiology, and lung lesions were scored based on the percentage of gross lesions in each of the lung lobes. The percentage for each lobe was summarized and then weighted for each lobe to give an overall score for each animal. Lung lobe weighting was predefined based on the ratio of individual lobes to total lung mass using slightly different criteria between the site in the Mhyop-only challenge (left apical 10%, left cardiac 10%, left diaphragmatic 25%, right apical 10%, right cardiac 10%, right diaphragmatic 25% and intermediate 8%) and the Mhyop+Pm dual challenge study (left apical 6%, left cardiac 10%, left diaphragmatic 31%, right apical 5%, right cardiac 10%, right diaphragmatic 30% and intermediate 8%), although both scoring systems had a maximum score possible of 100 and are comparable [28].

**Table 3:** System of categorisation of clinical scores in Mhyop+Pm dual-challenge study

Parameter	Score			
	0	1	2	3
Rectal temperature	37.5 °C – 39.5 °C	39.5 °C – 40.0 °C	> 40.0 °C – 40.9 °C	≥ 41.0 °C or < 37.5 °C
Demeanour	Normal	Mild Depression (reduced activity, reduced alertness)	Moderate Depression (reluctance to rise, reduced appetite)	Severe Depression (recumbence, moribund)
Nasal discharge	Absent	Serous	Seromucoid	Mucoid
Coughing	Absent	1–2 dry cough	> 2 dry cough or 1–2 productive coughs	> 2 productive coughs
Respiration	Normal	Mild (Slightly increased rate or effort)	Moderate (More pronounced increased rate and effort)	Severe (Respiratory distress, open mouth breathing)

**Table 4:** System of categorisation of clinical scores in *M. hyopneumoniae* alone challenge study and in both field studies

Score	Severity	Clinical findings	Clinically affected
0	Normal	No abnormal respiratory findings	No
1	Mild	Mildly abnormal character of respiration with or without other mild clinical signs	Yes, only if rectal T ≥ 40 °C
2	Moderate	Moderately abnormal character of respiration, may have mild depression and noticeable dyspnoea	Yes, independently of rectal T
3	Severe	Severely abnormal character of respiration, may have moderate depression and pronounced dyspnoea; mouth breathing may be observed.	Yes, independently of rectal T

A new clinical case was a healthy in contact pig on Day 0 that met the clinically affected criteria any time after Day 0 up to study end. In the field, a treatment success was a clinically affected pig on Day 0 no longer meeting the criteria on treatment completion and not relapsing up to study end

Lung lavage was collected from each animal at necropsy (after removal and scoring of lungs but before collection of tissue samples) using 50 ml sterile phosphate buffered saline (PBS) per pig. The PBS was introduced into the lungs via the trachea and the lungs massaged to ensure distribution of the PBS throughout the lungs. Lung fluid was then collected by inverting the lungs over a suitable sterile container. Lavage samples in the Mhyop-only study was submitted to PCR for *M. hyopneumoniae* as previously described [29]. In the Mhyop+Pm dual-challenge study, lung lavage was used for *M. hyopneumoniae* culture and lung tissue for *P. multocida*. For *M. hyopneumoniae* counts, lung lavage was placed in Mycoplasma broth and thereafter inoculated onto Mycoplasma agar plates. The resultant colonies were counted after incubation for 10 to 14 days. For *P. multocida*, lung samples were homogenised and the homogenate was diluted in peptone water. Aliquots of homogenate dilution were placed on the surface of 5% sheep blood agar plates and incubated overnight for 16 to 24 h.

### Field studies design

In the field studies, two commercial farms were selected, one in Hungary and one in France, where previous outbreaks of EP had been reported. Both farms vaccinated against *M. hyopneumoniae* and PCV2 at weaning. In addition, the site in Hungary also vaccinated against *Actinobacillus pleuropneumoniae* during the nursery period. After study enrolment, apart from vaccination, pigs in both sites did not receive any mass medication other than tylvalosin in water. Pigs from a single batch were randomised to pens on arrival at the fattening unit at approximately 11 weeks of age in both sites. At the onset of the EP outbreak (approximately 16 weeks of age in both sites), each water line was randomly assigned to either group A and given TVN, or group B in which pigs classed as clinically affected were treated with tylosin injections (TYL), whilst healthy in contact pigs were left unmedicated as controls (CTL) to allow metaphylaxis evaluation. Enrolled animals had no clinical signs of other respiratory, enteric or systemic disease.

#### Diagnosis of EP in the field outbreak

Prior to study start, the presence of EP in the enrolled pigs on each site was demonstrated by the presence of clinical signs and by isolation or PCR testing for specific bacteria in BAL from clinically affected pigs. To perform bronchoalveolar lavage washing, the animal was restrained with a rope over the maxilla. The pig’s mouth was be held open with a gag in order to insert the catheter between rope and palate in direction of pharynx. Lavage was collected with a sterile catheter. The catheter was deeply inserted into the trachea as the pig inspired, then rotated and moved up and down. Subsequently, 10 ml of 0.1 M PBS pH 7.4 containing 0.15 M NaCl will be introduced as deeply through the catheter and immediately aspirated. The recovered fluid was collected in sterile tubes.

Once at least 30% of pens had at least 10% of pigs in that pen classified as clinically affected, pneumonic lesions had been identified at necropsy and BAL samples from clinically affected pigs tested positive for *M. hyopneumoniae* by PCR, treatment of all clinically affected pigs and metaphylaxis of healthy in contact pigs commenced on Day 0. Pathogens involved in the outbreak were monitored by BAL collected from a selection of clinically affected pigs at the onset of the outbreak. The BAL samples were submitted to *M. hyopneumoniae* PCR and to bacteriology. The tip of the catheter (~ 1 cm) used for BAL collection served as and was used for *M. hyopneumoniae* and *M. hyorhinis* isolation. In addition, oral fluids collected from cotton ropes hung for 30 min in a selection of pens on Day 0 were used to monitor respiratory pathogens [30]. These oral fluid samples were submitted for PCR testing for *M. hyopneumoniae*, porcine reproductive and respiratory syndrome (PRRS) virus, porcine circovirus type 2 (PCV2) and swine influenza virus (SIV). *M. hyopneumoniae* PCR in Hungary was conducted using a commercial kit (BactoReal®, Ingenetix) and in France using methodology as previously described [31]. Tracheobronchial swabs were collected from clinically affected pigs around Day 0 and at intervals during the study for *M. hyopneumoniae* isolation.

At both study sites, the samples indicated high prevalence of *M. hyopneumoniae* and *P. multocida* (Table 5). In addition, field isolates of *M. hyopneumoniae* had MICs to tylvalosin of 0.008 μg/ml from the site in Hungary and ≤ 0.002 μg/ml from the site in France for tylvalosin and values of 0.06 and 0.03 μg/ml, respectively, for the same isolates to tylosin.

**Table 5:** Frequency of bacterial isolation and *M. hyopneumoniae* presence by isolation or by PCR tests from bronchoalveolar lavage fluid or tracheobronchial swab samples before treatment initiation

Study site	Number of animals sampled (***n***)	Positive Isolation (***n***)^**a**^							Positive ***Mhyop*** PCR (***n***)^**b**^
		***APP***	***Gla***	***Ssuis***	***Pm***	***Bb***	***Mhyor***	***Mhyop***	
**Hungary**	13	0	1	0	4	0	11	0	10
**France**	15	0	11	14	8	6	15	1	4

*Gla Glässerella parasuis*, *Pm Pasteurella multocida*, *Mhyor Mycoplasma hyorhinis*, *Mhyop Mycoplasma hyopneumoniae*, *Bb Bordetella bronchiseptica*, *APP Actinobacillus pleuropneumoniae*, *Ssuis Streptococcus suis*

^a^Isolation of pathogens was from BALF samples except for *Mhyor* and *Mhyop* which were from TBS

^b^PCR was performed on BALF samples

Other bacteria also detected in BAL included *Bordetella bronchiseptica*, *Glässerella parasuis*, *Streptococcus suis* and *Mycoplasma hyorhinis* (Table 5). Results from PCR performed on oral fluids on treatment initiation day, showed that 5 of 21 pens in Hungary were positive for *M. hyopneumoniae* and all were negative for relevant viruses. In France, oral fluids were positive by PCR for PRRS virus in 30 of 36 pens and SIV in 22 of 36 pens on treatment initiation day and were negative for *M. hyopneumoniae*.

#### Assessment of treatment efficacy in field studies

Treatment efficacy was measured in terms of cure rates at treatment completion and relapse from completion of the course of medication (Day 3 or 5) through to study completion (Day 13). This variable was measured as those pigs that were clinically affected on Day 0, but no longer clinically affected (as categorised by the specified criteria, Table 4) on treatment completion, and that did not relapse to study end on Day 13. Animals in either group that relapsed after completion of the course of treatment were medicated with tulathromycin as rescue therapy.

#### Assessment of metaphylactic efficacy in field studies

In both field studies, metaphylaxis efficacy was evaluated by measuring the incidence of new clinical cases of respiratory disease in the healthy population of pigs (Table 2). Healthy in contact pigs in the TVN pens received the same medication as the pigs enrolled in the treatment efficacy evaluation. Healthy in contact pigs in the TYL pens were left untreated and served as metaphylaxis control (CTL) group.

Metaphylactic efficacy was evaluated from tylvalosin treatment completion (Day 5) through to study completion (Day 13). Animals in both groups that were healthy on Day 5 but met the clinically affected criteria (Table 4) up to Day 13 were classed as new clinical cases and were medicated with tulathromycin as rescue therapy.

### Data analysis

In these studies, the experimental unit was the pen. When the individual pig was the unit of analysis, random effect of pen was included in the models when possible. The data were analysed using SAS® Version 9.4 (SAS Institute). The primary efficacy criterion of both challenge studies was pen arithmetic mean lung lesion score for the pigs in a pen and was analysed using a general linear model (GLM). Pen coughing score in the Mhyop-only challenge study was analysed as the percentage of pig-alive days in which pigs were observed to be coughing (range from 0 to 100%), using the same model and procedure as that for pen mean lung lesion score. Pen mean daily clinical scores were evaluated using a GLM. Pen mean *M. hyopneoumiae* genomic copies in the Mhyop-only study were analysed using GLM after log transformation.

In the Mhyop+Pm dual-challenge study, *M. hyopneumoniae* counts recovered from lung lavage of individual pigs was analysed by SAS Mixed Procedure and *P. multocida* counts recovered from lung tissue with a GLM. For both, the pen mean count was calculated and log transformed and compared between groups. The proportion of samples positive to either pathogen in the dual-challenge study was analysed using a logistic model. Data of field studies were analysed separately for each site and further evaluated in a multi-centre analysis. In the field studies, the primary variable for treatment efficacy was ‘treatment success’ for pigs clinically affected on Day 0. This was a binary variable where the proportion of treatment success pigs was evaluated using a logistic model with random effect of pen in individual sites, and random effect of site for the multi-centre analysis. In addition, treatment success was evaluated with a non-inferiority test (Farrington Manning) by comparing TVN and TYL (pre-defined non-inferiority margin 20%). Evaluation of metaphylactic efficacy was evaluated by comparison of the primary variable incidence of new cases of respiratory disease in the healthy population from treatment completion to study end. This was a binary variable where the incidence of new cases of respiratory disease was evaluated using a logistic model with random effect of pen in individual sites and random effect of site for the multi-site analysis. In addition, disease incidence data were analysed using survival analysis on data of both sites combined.

## Data Availability

Due to confidentiality agreements with research collaborators and commercially sensitive nature of the research, data are subject to access restriction. Please contact the corresponding author for any request.

## Abbreviations

APP: *Actinobacillus pleuropneumoniae*
BAL: Bronchoalveolar lavage
CTL: Control
DNA: Deoxyribonucleic acid
e.g.: For example
ELISA: Enzyme linked immunosorbent assay
EP: Enzootic pneumonia
GLM: General linear model
IM: Intramuscular
log: Logarithm
Mhyop: *M. hyopneumoniae*
Mhyop+Pm: *M. hyopneumoniae* + *P. multocida*
MIC: Minimum inhibitory concentration
PBS: Phosphate buffered saline
PCR: Polymerase chain reaction
PCV2: Porcine circovirus type 2
PRRS: Porcine reproductive and respiratory syndrome
SAS: Statistical analysis software (SAS Institute, North Carolina)
SD: Standard deviation
SIV: Swine influenza virus
TVN: Tylvalosin
TYL: Tylosin
vs.: Versus

## References

[1] Maes D, Sibila M, Kuhnert P, Segales J, Haesebrouck F, Pieters M (2018). Update on mycoplasma hyopneumoniae infections in pigs: knowledge gaps for improved disease control. Transbound Emerg Dis.

[2] Pieters MG, Maes D, Zimmerman JJ, Karriker LA, Ramirez A, Schwartz KJ, Stevenson GW, Zhang J (2019). Mycoplasmosis. Diseases of swine.

[3] CVMP European public assessment report (EPAR) for Aivlosin [Available from: https://www.ema.europa.eu/en/documents/product-information/aivlosin-epar-product-information_en.pdf].

[4] Nanjiani IA, McKelvie J, Benchaoui HA, Godinho KS, Sherington J, Sunderland SJ (2005). Evaluation of the therapeutic activity of tulathromycin against swine respiratory disease on farms in Europe. Vet Ther.

[5] Vicca J, Maes D, Jonker L, de Kruif A, Haesebrouck F (2005). Efficacy of in-feed medication with tylosin for the treatment and control of mycoplasma hyopneumoniae infections. Vet Rec.

[6] Morrison RB, Pijoan C, Hilley HD, Rapp V (1985). Microorganisms associated with pneumonia in slaughter weight swine. Can J Comp Med.

[7] McKelvie J, Morgan JH, Nanjiani IA, Sherington J, Rowan TG, Sunderland SJ (2005). Evaluation of tulathromycin for the treatment of pneumonia following experimental infection of swine with mycoplasma hyopneumoniae. Vet Ther.

[8] Del Pozo SR, Thiry J, Vranckx K, Lopez Rodriguez A, Chiers K, Haesebrouck F (2012). Efficacy of florfenicol injection in the treatment of mycoplasma hyopneumoniae induced respiratory disease in pigs. Vet J.

[9] Pieters M, Fano E, Pijoan C, Dee S (2010). An experimental model to evaluate mycoplasma hyopneumoniae transmission from asymptomatic carriers to unvaccinated and vaccinated sentinel pigs. Can J Vet Res.

[10] Amass SF, Clark LK, van Alstine WG, Bowersock TL, Murphy DA, Knox KE (1994). Interaction of mycoplasma hyopneumoniae and Pasteurella multocida infections in swine. J Am Vet Med Assoc.

[11] Ciprián A, Pijoan C, Cruz T, Camacho J, Tórtora J, Colmenares G (1988). Mycoplasma hyopneumoniae increases the susceptibility of pigs to experimental Pasteurella multocida pneumonia. Can J Vet Res.

[12] Garcia-Morante B, Segalés J, López-Soria S, de Rozas AP, Maiti H, Coll T (2016). Induction of mycoplasmal pneumonia in experimentally infected pigs by means of different inoculation routes. Vet Res.

[13] Garcia-Morante B, Segalés J, Serrano E, Sibila M (2017). Determinants for swine mycoplasmal pneumonia reproduction under experimental conditions: a systematic review and recursive partitioning analysis. PLoS One.

[14] Tavío MM, Poveda C, Assunção P, Ramírez AS, Poveda JB (2014). In vitro activity of tylvalosin against Spanish field strains of mycoplasma hyopneumoniae. Vet Rec.

[15] Lees P, Illambas J, Potter TJ, Pelligand L, Rycroft A, Toutain PL (2017). A large potentiation effect of serum on the in vitro potency of tulathromycin against Mannheimia haemolytica and Pasteurella multocida. J Vet Pharmacol Ther.

[16] McClary DG, Loneragan GH, Shryock TR, Carter BL, Guthrie CA, Corbin MJ (2011). Relationship of in vitro minimum inhibitory concentrations of tilmicosin against Mannheimia haemolytica and Pasteurella multocida and in vivo tilmicosin treatment outcome among calves with signs of bovine respiratory disease. J Am Vet Med Assoc.

[17] Benchaoui HA, Nowakowski M, Sherington J, Rowan TG, Sunderland SJ (2004). Pharmacokinetics and lung tissue concentrations of tulathromycin in swine. J Vet Pharmacol Ther.

[18] Moges R, De Lamache DD, Sajedy S, Renaux BS, Hollenberg MD, Muench G (2018). Anti-inflammatory benefits of antibiotics: Tylvalosin induces apoptosis of porcine neutrophils and macrophages, promotes Efferocytosis, and inhibits pro-inflammatory CXCL-8, IL1α, and LTB4 production, while inducing the release of pro-resolving Lipoxin A4 and Resolvin D1. Front Vet Sci.

[19] Tisoncik JR, Korth MJ, Simmons CP, Farrar J, Martin TR, Katze MG (2012). Into the eye of the cytokine storm. Microbiol Mol Biol Rev.

[20] Meyns T, Maes D, Dewulf J, Vicca J, Haesebrouck F, de Kruif A (2004). Quantification of the spread of mycoplasma hyopneumoniae in nursery pigs using transmission experiments. Prev Vet Med.

[21] Meyns T, Dewulf J, de Kruif A, Calus D, Haesebrouck F, Maes D (2006). Comparison of transmission of mycoplasma hyopneumoniae in vaccinated and non-vaccinated populations. Vaccine..

[22] Holst S, Yeske P, Pieters M (2020). Elimination of mycoplasma hyopneumoniae from breed-to-wean farms: a review of current protocols with emphasis on herd closure and medication. J Swine Health Prod.

[23] Pieters M, Daniels J, Rovira A (2017). Comparison of sample types and diagnostic methods for *in vivo* detection of mycoplasma hyopneumoniae during early stages of infection. Vet Microbiol.

[24] CVMP Guidance for Industry #85 / VICH GL 9 Good Clinical Practice (GCP), CVMP Guideline for the demonstration of efficacy for veterinary medicinal products containing antimicrobial substances. EMA/CVMP/627/2001-Rev.1. 2016 [Available from: https://www.ema.europa.eu/en/documents/scientific-guideline/vich-gl9-good-clinical-practices-step-7_en.pdf].

[25] Bereiter M, Young TF, Joo HS, Ross RF (1990). Evaluation of the ELISA and comparison to the complement fixation test and radial immunodiffusion enzyme assay for detection of antibodies against mycoplasma hyopneumoniae in swine serum. Vet Microbiol.

[26] Hannan PC (2000). Guidelines and recommendations for antimicrobial minimum inhibitory concentration (MIC) testing against veterinary mycoplasma species. International research programme on comparative mycoplasmology. Vet Res.

[27] Tanner AC, Wu CC (1992). Adaptation of the sensititre broth microdilution technique to antimicrobial susceptibility testing of mycoplasma gallisepticum. Avian Dis.

[28] Garcia-Morante B, Segalés J, Fraile L, Pérez de Rozas A, Maiti H, Coll T (2016). Assessment of mycoplasma hyopneumoniae-induced pneumonia using different lung lesion scoring systems: a comparative review. J Comp Pathol.

[29] Strait EL, Madsen ML, Minion FC, Christopher-Hennings J, Dammen M, Jones KR (2008). Real-time PCR assays to address genetic diversity among strains of mycoplasma hyopneumoniae. J Clin Microbiol.

[30] Hernandez-Garcia J, Robben N, Magnée D, Eley T, Dennis I, Kayes SM (2017). The use of oral fluids to monitor key pathogens in porcine respiratory disease complex. Porc Health Manag.

[31] Marois C, Dory D, Fablet C, Madec F, Kobisch M (2010). Development of a quantitative real-time TaqMan PCR assay for determination of the minimal dose of mycoplasma hyopneumoniae strain 116 required to induce pneumonia in SPF pigs. J Appl Microbiol.

